# Artificial Intelligence Applied in Early Prediction of Lower Limb Fracture Complications

**DOI:** 10.3390/clinpract14060197

**Published:** 2024-11-14

**Authors:** Aurelian-Dumitrache Anghele, Virginia Marina, Liliana Dragomir, Cosmina Alina Moscu, Iuliu Fulga, Mihaela Anghele, Cristina-Mihaela Popescu

**Affiliations:** 1 Doctoral School, “Dunărea de Jos” University, 800201 Galati, Romania; anghele_aurelian@yahoo.com (A.-D.A.); cosmina_caluian@yahoo.com (C.A.M.); 2 Medical Department of Occupational Health, Faculty of Medicine and Pharmacy, “Dunărea de Jos” University, 800201 Galati, Romania; 3 Clinical-Medical Department, Faculty of Medicine and Pharmacy, “Dunărea de Jos” University, 800201 Galati, Romania; lilianadragomir2017@gmail.com (L.D.); mihaela.anghele@ugal.ro (M.A.); 4 Department of General Surgery, Faculty of Medicine and Pharmacy, “Dunărea de Jos” University, 800201 Galati, Romania; iuliu.fulga@ugal.ro; 5 Dental-Medicine Department, Faculty of Medicine and Pharmacy, “Dunărea de Jos” University, 800201 Galați, Romania; cristina.popescu@ugal.ro

**Keywords:** thrombosis, venous thrombosis, femur fracture, artificial intelligence

## Abstract

**Background**: Artificial intelligence has become a valuable tool for diagnosing and detecting postoperative complications early. Through imaging and biochemical markers, clinicians can anticipate the clinical progression of patients and the risk of long-term complications that could impact the quality of life or even be life-threatening. In this context, artificial intelligence is crucial for identifying early signs of complications and enabling clinicians to take preventive measures before problems worsen. **Materials and methods***:* This observational study analyzed medical charts from the electronic archive of the Clinical Emergency Hospital in Galați, Romania, covering a four-year period from 2018 to 2022. A neural network model was developed to analyze various socio-demographic and paraclinical data. Key features included patient demographics, laboratory investigations, and clinical outcomes. Statistical analyses were performed to identify significant risk factors associated with deep venous thrombosis (DVT). **Results***:* The analysis revealed a higher prevalence of female patients (60.78%) compared to male patients, indicating a potential gender-related risk factor for DVT. The incidence of DVT was highest among patients aged 71 to 90 years, affecting 56.86% of individuals in this age group, suggesting that advanced age significantly contributes to the risk of developing DVT. Additionally, among the DVT patients, 15.69% had a body mass index (BMI) greater than 30, categorizing them as obese, which is known to increase the risk of thrombotic events. Furthermore, this study highlighted that the highest frequency of DVT was associated with femur fractures, occurring in 52% of patients with this type of injury. The neural network analysis indicated that elevated levels of direct bilirubin (≥1.5 mg/dL) and prothrombin activity (≤60%) were strong predictors of fracture-related complications, with sensitivity and specificity rates of 78% and 82%, respectively. These findings underscore the importance of monitoring these laboratory markers in at-risk populations for early intervention. **Conclusions***:* This study identified critical risk factors for developing DVT, including advanced age, high BMI, and femur fractures, which necessitate longer recovery periods. Additionally, the findings indicate that elevated direct bilirubin and prothrombin activity play a significant role in predicting DVT development. These results suggest that AI can effectively enhance the anticipation of clinical evolution in patients, aiding in early intervention and management strategies.

## 1. Introduction

In trauma patients, especially those with lower extremity fracture complications following the traumatic event are common. Immediate complications typically involve critical issues such as airway management, breathing difficulties, or considerable blood loss. These require urgent intervention to stabilize the patient and prevent life-threatening outcomes. These complications significantly impact patient outcomes and often require prompt intervention to prevent life-threatening consequences. Despite advancements in trauma care, traditional methods of monitoring and managing these complications rely largely on clinical assessment and retrospective data analysis, which may delay detection and limit preventive measures [1]. Less immediate complications that arise after a fracture include acute compartment syndrome and fat embolism syndrome, both of which can develop after the acute phase [1]. Other complications that may occur during the recovery process include pressure injuries, autonomic dysreflexia, pain, muscle spasms, heterotopic ossification, and nonunion of the fracture [2].

An important and potentially life-threatening complication in trauma patients is venous thromboembolism (VTE), which can develop both early, within the first 48 h, and later, after one week or even after two weeks post-injury [3,4,5]. Trauma patients, regardless of age, have a heightened risk of VTE—estimated to be 13 times higher than non-trauma patients—largely due to immobilization and factors outlined in Virchow’s triad [6,7,8,9]. Both major and minor trauma pose a significant risk for DVTs due to immobilization and anatomic factors [10]. Venous thromboembolism commonly manifests as deep vein thrombosis (DVT) or pulmonary thromboembolism (PTE) [11,12].

According to recent studies on the distribution of deep vein thrombosis in lower extremity fractures, the highest incidence of fractures was identified in hip fractures (17–58%), while the incidence of corrected intra-operatively distal femur fractures was 25%, and the incidence in foot and ankle surgery was 2.1% [13]. These studies vary across medical centers, where differences in prophylactic therapy and study characteristics have resulted in varied DVT incidence rates.

The risk of DVT is significantly elevated in patients undergoing major orthopedic or neurovascular surgeries, particularly those with risk factors like advanced age, previous DVT history, and other medical conditions. Prolonged surgical times and post-surgical immobilization further exacerbate this risk, with a 4-year recurrence rate for surgically induced DVTs between 5% and 11% depending on the procedure [14]. Additionally, long-duration travel and immobilization due to conditions like hemiplegia after a stroke also increase the risk of DVT [15].

Artificial intelligence (AI) introduces a novel approach to addressing these challenges by enabling proactive, real-time prediction of complications. By analyzing imaging data and patient-specific information, such as age, BMI, and medical history, AI algorithms can anticipate complications like DVT and pulmonary embolism before they become clinically apparent. This predictive capability is particularly valuable in trauma settings, where early intervention can dramatically improve patient outcomes.

Artificial intelligence (AI) has shown great potential in supporting the detection and management of complications associated with lower limb fractures, such as DVT, pulmonary embolism, and acute compartment syndrome. By analyzing imaging data, AI algorithms can identify early signs of bone density changes, compartment syndrome, or abnormal fracture healing, which may lead to complications. Additionally, AI can assist in predicting high-risk cases of venous thromboembolism (VTE) by integrating patient data like age, BMI, and medical history, which are known risk factors. Continuous monitoring of vital signs through AI can also enable the early detection of life-threatening conditions, such as respiratory compromise in patients at risk for pulmonary embolism. In these ways, AI serves as a valuable tool for both early intervention and personalized management, aiming to improve outcomes and reduce the burden of complications in trauma patients.

The main contribution of this study is the development of a neural network-based AI model that integrates and analyzes multiple risk factors to identify patients at high risk for complications associated with lower limb fractures. This model not only enhances the accuracy of complication prediction but also allows for more personalized and timely management, representing a substantial innovation in trauma care.

## 2. Materials and Methods

The data extraction process for this observational and longitudinal retrospective cohort study involved systematically reviewing medical charts archived electronically at the Clinical Emergency Hospital in Galați, Romania. Medical records of patients hospitalized between January 2018 and December 2022 were examined to create a comprehensive database. Each chart was assessed according to the study’s inclusion criteria—specifically, patients over 18 years old hospitalized for a lower leg fracture.

The absence of exclusion criteria in this study was intentional to ensure the inclusivity and generalizability of the findings. By not excluding patients based on additional medical or demographic factors, this study encompasses a broader, more representative sample of individuals who experience lower leg fractures. This inclusive approach allows for a comprehensive analysis of the incidence and risk factors associated with complications, such as deep vein thrombosis (DVT), across diverse patient profiles. Consequently, the results are more reflective of real-world conditions, enhancing the study’s external validity and providing insights applicable to a wide range of clinical settings.

Ethical approval for this study was obtained from the Ethics Committee of the Clinical Emergency Hospital in Galați, Romania, ensuring compliance with ethical standards for research involving human subjects.

To ensure consistency, the team used a standardized data extraction form. Trained personnel documented key variables such as age, gender, fracture location, BMI, lab results, smoking status, history of venous thrombosis, stroke, and hormonal therapy use. This data was entered into a structured database designed for accuracy and uniformity across entries. Quality checks, including cross-verifying randomly selected entries, helped maintain data reliability. This rigorous approach enabled an accurate analysis of the incidence and risk factors associated with complications like DVT among the cohort.

The incidence rate of DVT among patients with lower limb fractures was 171 cases per 1000 patients (51 out of 299 patients).

This incidence rate highlights a significant risk for complications in this patient population, necessitating vigilant monitoring and prophylactic measures to mitigate the potential impact of DVT.

**Figure 1:**
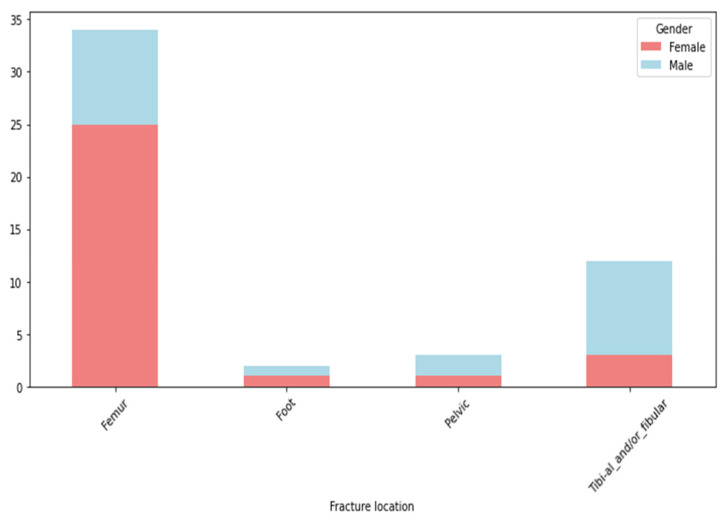
Patients who developed DVT during hospitalization.

They represent the DVT sub-group and are presented in Figure 1.

All patient data were included in a Microsoft Excel table, and statistical analysis was performed using IBM SPSS 29.0.2.0. Easy NN-plus (version 14.0 -16.0.0.1) for Windows was employed to develop neural networks.

In the analysis, specific statistical methods applied using IBM SPSS included descriptive statistics to summarize demographic and clinical characteristics of the cohort, such as mean, median, standard deviation, and frequency distributions. Comparative analyses were conducted to assess differences between groups (e.g., patients with and without DVT), using tests such as the Chi-square test for categorical variables and *t*-tests or ANOVA for continuous variables. Additionally, logistic regression analysis was employed to identify potential risk factors associated with the development of DVT, controlling for variables like age, BMI, and fracture location. These statistical methods provided a comprehensive understanding of the factors influencing complication rates in the patient cohort.

The neural networks in our AI algorithm were developed using the Easy NN-Plus software, following a structured process to optimize accuracy and predictive capability. Input data, including patient demographics, fracture details, and lab results, were pre-processed and standardized before training. Key hyper-parameters were systematically tuned to optimize the model’s predictive capability. Specifically, the architecture was iteratively adjusted by varying the number of hidden layers and neurons to balance accuracy with computational efficiency.

The network architecture was determined through iterative testing to establish the optimal number of layers and nodes, balancing complexity with computational efficiency.

During model training, a range of learning rates was tested to identify the optimal value for backpropagation, ensuring that the network effectively minimized prediction error without overfitting. Hyper-parameter optimization also included evaluating different batch sizes and epochs to maximize the model’s stability and performance. To validate the model, we employed cross-validation, partitioning the dataset into training and test sets. Model performance was assessed using metrics such as sensitivity, specificity, and accuracy, ensuring robust validation of the network configuration before deployment. The algorithm’s predictive accuracy was then assessed using performance metrics such as sensitivity, specificity, and accuracy, ensuring that the model reliably identified key risk factors for complications like DVT within the patient cohort.

This study aims to highlight variables associated with a high risk of developing DVT following a lower limb fracture using an artificial intelligence algorithm based on neural networks. Patients or their relatives gave informed consent for their data to be included in this study.

## 3. Results

This study focuses on identifying variables that can predict, early on, which patients will develop DVT following hospitalization for lower limb fractures.

**Figure 2:**
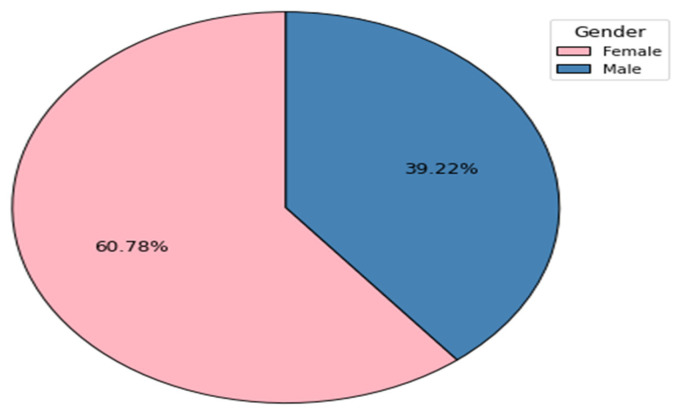
Gender distribution of DVTs.

In the DVT subgroup, there was a difference of more than 20% between women and men. Females represented 60.78% (n = 31), while 39.22% (n = 20) were male (Figure 2).

Age distribution in the DVT patients is represented in Table 1.

The average age of the DVT patients was 71.65 years, with a standard deviation of 16.238. The median age was 77, with the youngest subject 32 and the oldest 97. Table 1 summarizes these data.

**Figure 3:**
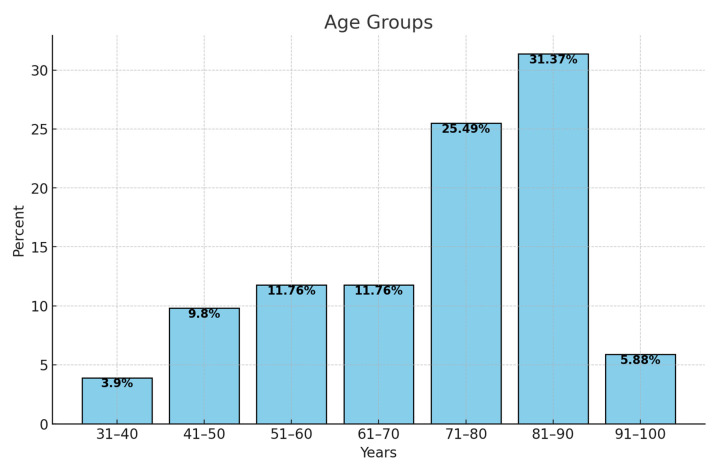
Distribution of patients by age.

Given that the risk of developing DVT increases with age [16], we grouped the patients into age groups, each one corresponding to a decade. The following percentages associated with each group were identified (Figure 3).

The highest percentages were identified among patients aged 81 to 90, i.e., 31.4% (n = 16). Patients aged 71 to 80 years accounted for 25.5% (n = 13), patients aged 51 to 60 years and 61 to 70 each accounted for 11.8% (n = 6) of all DVTs, and those aged between 41 and 50 years accounted for 9.8% (n = 5) of all DVTs. Patients in the 91–100 age group accounted for 5.9% (n = 3) of this group. Only 3.9% (n = 2) were in the age group 31–40 years.

**Figure 4:**
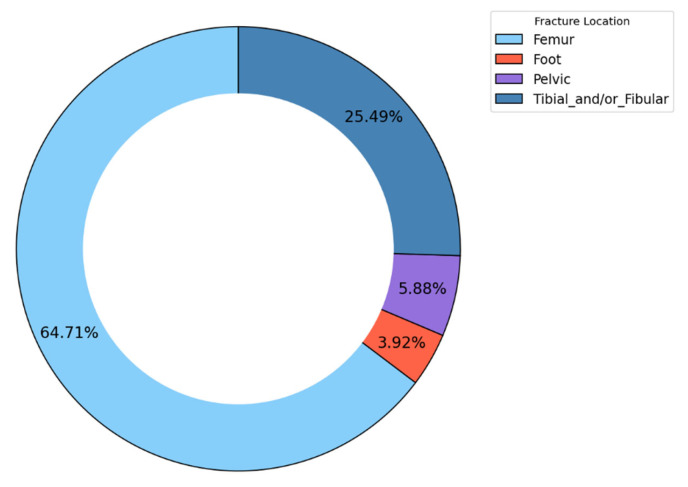
Fracture location distribution in DVT patients.

DVT developed in a subgroup of patients admitted for lower limb fractures. The distribution of these fractures is shown in Figure 4.

**Figure 5:**
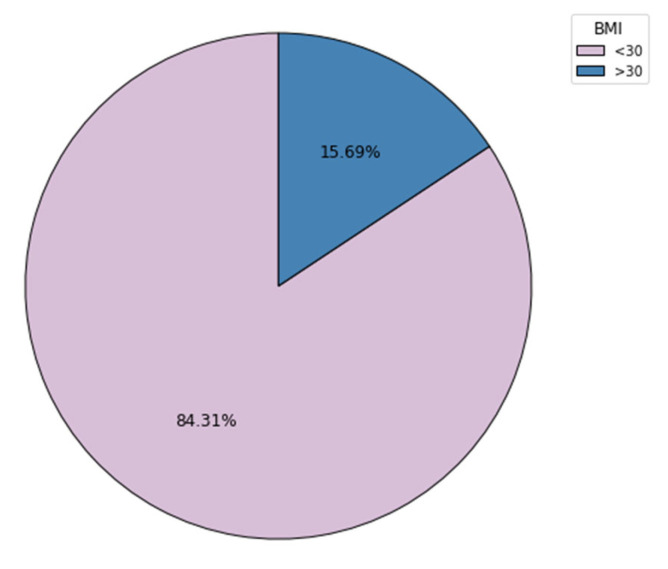
BMI over thirty distributions in the DVT cohort.

More than 60% of the DVT patients in our cohort had femur fractures (64.71%; n = 33), 25.49% (n = 13) had tibial and/or fibular fractures, and pelvic fractures accounted for 5.88% (n = 3) of all fractures. Foot fractures were present in 3.92% (n = 2) of patients. Data is represented in Figure 5.

An important risk factor for developing DVT is obesity, defined as BMI over 30 kg/m^2^. In our cohort of patients who had DVT, 15.69% (n = 8) had a BMI over 30, and 84.31% did not.

**Figure 6:**
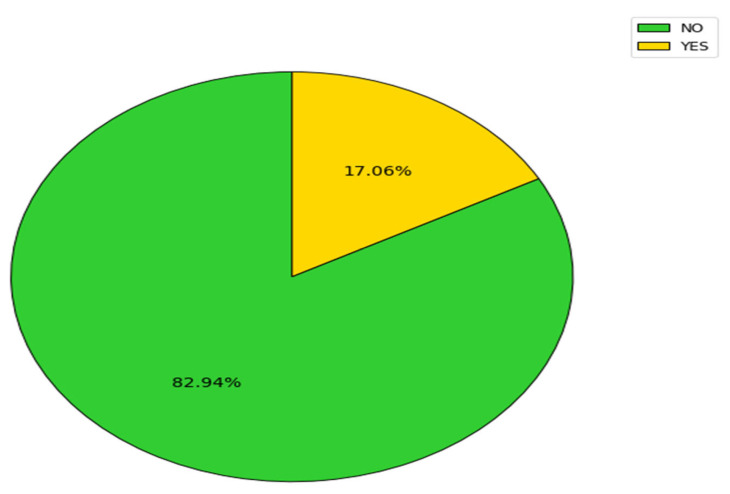
DVT percentage in patients with lower limb fracture.

Considering the 299 group of patients who were admitted for lower limb fracture, under a fifth developed DVT (Figure 6).

**Figure 7:**
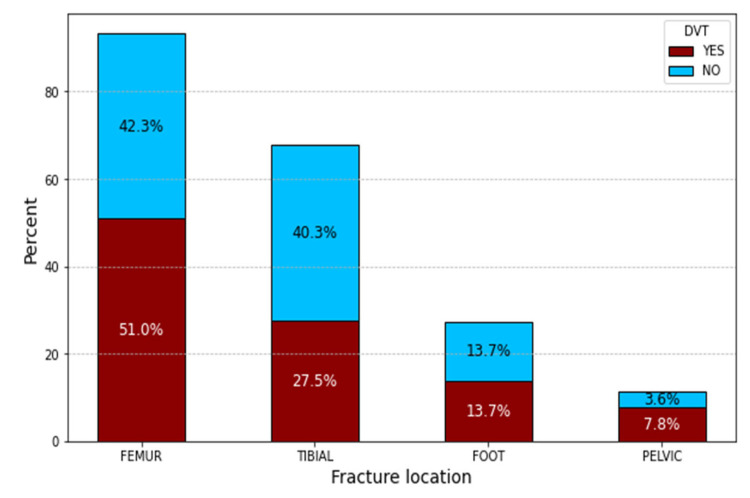
DVT percentage in different fracture locations.

The femur had the highest percentage of fracture locations associated with DVT, followed by tibial and/or fibular and pelvic fractures. Foot fractures had the smallest percentage of DVT, as shown in Figure 7.

The use of artificial intelligence in the early detection of lower limb fracture complications can bring numerous benefits, including:

- Early detection of complications: Artificial intelligence can help identify early signs of complications, enabling doctors to intervene before problems worsen.
- Personalizing treatment: Artificial intelligence identifies individual risk factors and tailors treatment to each patient’s needs.
- Increasing efficiency: Artificial intelligence can reduce the time it takes to detect complications and improve treatment efficiency.

A neural network (NN)-based model was constructed to identify and analyze the links between the specified medical analysis data and health status.

The Easy NN program was utilized, and the following steps were taken: determination of input data, establishing the optimal architecture, network training, and using the neural model to analyze the influence of input on output, optimize output values, and predict output values. The input data chosen were extracted from archived medical charts of patients admitted for lower limb fractures from 2018 to 2022, including personal information and specific medical test results related to venous thrombosis (Table 2).

After analyzing all the data using the neural network, the following results were obtained in Table 3:

For the “FALSE” health status, five out of the eight parameters display values outside normal limits. The first two parameters of importance are direct bilirubin and prothrombin activity. For the “TRUE” condition, only four of the eight parameters show abnormal values, with the first two falling within normal limits.

As a result of this analysis, a query of the neural model is necessary to optimize the health status while adhering to normal limits. To achieve this, the means of the normal ranges were applied to the inputs with values outside the normal ranges, and the query was conducted to determine the TRUE state. The outcomes are presented in Table 4.

Table 4 shows that the imposed values do not affect the previously optimized values of direct bilirubin and prothrombin activity, confirming that these parameters significantly influence health status.

## 4. Discussion

Trauma is a leading cause of mortality and disability all over the world [17]. Trauma affects not only the body but also the psyche to the extent that depends on the genetic traits of each individual and their coping mechanisms [18]. Whether head, abdominal, or orthopedic trauma, such events predispose patients to sustain VTE, which is an essential factor adding to mortality and morbidity [9].

Specialized studies in the United States show that this condition has an annual incidence of 80 reported cases per hundred thousand [19]. Statistics in the United States indicate that more than 200,000 people risk developing venous thrombosis yearly. Of these cases, approximately 25% are at risk of complications from pulmonary embolism [20].

In England, the number of deaths from VTE is higher than the total number of deaths from breast cancer, AIDS, and road traffic accidents, and more than twenty-five times the number of deaths from MRSA [21]. The numbers are alarmingly high, but more alarming is the fact that many of these deaths are preventable. There is a safe, effective, and cost-effective method of preventing venous thrombosis that is not as widely administered as it should be. Worldwide, VTE occupies the third place when it comes to common death causes [22].

In the last decade, the advancement of imaging diagnostic tools led to an increased number of thromboembolic events being diagnosed in preterm infants. Also, therapeutic improvements in supportive neonatal intensive care units increased the number of preterm patients with thromboembolic risk factors who survived [23]. Such small patients spend a considerable amount of time hospitalized when indwelling catheters and sometimes sepsis, combined with the disruption of the delicate balance of homeostasis, lead to an increased risk of thromboembolism [23]. Although thromboembolic events in patients this age can have serious consequences, they are rare complications [24]. Unfortunately, few studies focus on pharmacotherapy, doses, and treatment duration in such small patients, and all mentioned before are extrapolated from adult case management [25].

Tibial shaft fractures account for 15% of pediatric long-bone fractures in the United States, making them the second most common pediatric trauma injury [26]. These fractures are typically treated with closed reduction and cast immobilization. However, in cases of open, unstable fractures with multiple or neurovascular injuries, surgical intervention may be necessary for stable internal and external fixation. This type of intervention requires limb immobilization until the fracture heals, so the intervention and postoperative management should be carefully chosen to prevent DVT.

Assessing the risk of developing DVT is a primary goal in surgical practice, and DVT prophylaxis plays a crucial role in managing trauma patients or those undergoing complex surgeries (Table 5).

While anticoagulant medication is essential in interventional practice, it is associated with the risk of major bleeding (MB) that can lead to hypovolemic shock and potentially lethal consequences.

Using non-vitamin K oral anticoagulants (NOACs) as anti-thrombotic drugs offers a viable replacement for vitamin K antagonists (VKAs) when preventing VTE recurrence, fatal PE, and long-term complications [27]. Dabigatran, rivaroxaban, apixaban, and edoxaban have been approved for treating acute VTE in large phase III trials [28,29] and have shown similar efficacy and a superior safety profile compared to VKAs [27,30,31].

The risk–benefit assessment can be conducted through a thorough medical history and additional tests to identify other associated risk factors.

Clinical diagnosis of DVT is often inaccurate [19]. If located close to the iliac vein, the pain can mimic appendicitis or adnexal pathology; the management team for such cases should include a general surgeon and a gynecologist [32,33].

Various probability scores have been developed to determine the likelihood of diagnosing DVT in the lower limb, with the Wells score (Table 6) being the most well-known.

DVT is unlikely if the Wells score is below 2 points, and the D-dimer testing is the next step. DVT is possible if the score is two or more, and further imaging exploration is recommended. For DVT, venous ultrasound is the primary diagnosing tool [35]. Although computer tomography (CT) is the gold standard for assessing many acute pathologies, i.e., head trauma [36], angiography CT is usually performed for PTE. CT venography may help determine the extent of a caval or iliac thrombosis [35]. Magnetic resonance direct thrombus imaging (MRDTI) is a possible option for investigating DVT, but it is not easily accessible and would increase costs [36]. Also, technical and logistical limitations associated with performing MRI of other areas (such as head MRI [37] apply here, making it less used as an imaging tool for lower leg DVT [38].

Incidence rates of VTE in men and women vary with age, and differences in the age range of the populations under study likely explain discrepancies in study results for overall VTE in men versus women [39,40,41,42,43]. In younger age groups (<50 years), the incidence of VTE is higher in women than in men [42,43,44] due to female reproductive risk factors (e.g., oral contraceptives and pregnancy), while in middle-aged persons (50–70 years), the incidence is higher in men than in women [40,44].

Although studies show a higher male prevalence of DVT, in our cohort, female patients accounted for more than 60%.

Venous thrombosis has a low rate in the first three decades of life, at about 1 per 10,000 cases per year. The incidence increases with age, especially after 45 years, reaching the peak of 5–6 per 1000 annually around 80 [40]. Tsai et al. reported in their study in 2002 that patients aged 85 or older had a 15 times higher risk of developing DVT than those aged 45–54 [45]. In our cohort, the greatest incidence of DVT was found in patients aged 81–90.

A critical modifiable risk factor for thrombosis is obesity, a body mass index (BMI) above 30 kg/m^2^ [46]. Obesity leads to a 2 to 3-fold higher risk of venous thrombosis in both men and women [45,47,48]. In our patients, obesity was present in eight.

The risk of thrombosis following surgery varies depending on the type of surgery and patient characteristics [40]. Interestingly, one study reported that older patients did not have a higher risk of postoperative venous thrombosis than younger patients for certain types of surgery [49,50].

Immobility increases the risk of thrombosis, likely due to the stagnation of blood flow in the venous system. Causes for immobility include bedrest, plaster casts on the lower limbs, and leg paralysis due to neurological/neurosurgical conditions. Research-based definitions of immobility due to bedrest vary, but four days seem reasonable. Minor forms of immobility, such as after minor surgery or injury, have also been associated with an increased risk of thrombosis [51]. This explains the prevalence of VTE in patients with femur fractures, as their recovery time is prolonged, and mobility is limited even after healing. The personality of individuals is a predictive factor for the occurrence of stress or burnout syndrome [52].

The goal of pharmacotherapy for patients is to reduce morbidity and prevent complications [53]. The rapid and comprehensive assessment of injuries is crucial in managing each case [21]. The prognosis largely depends on the presentation time to the doctor and the patient’s comorbidities [54]. There are both similarities and discrepancies between our cases and the literature. The vulnerabilities of the subjects, identified personality traits, and gender and age differences are all factors that play a role [55,56].

## 5. Conclusions

Our study identified risk factors for developing DVT, such as older age, high BMI, and femur fractures that require a longer recovery time. Additionally, this study found that elevated levels of direct bilirubin and prothrombin activity may play a predictive role in developing DVT. This suggests that artificial intelligence can help foresee patients’ clinical evolution.

### 5.1. Limitations

This study’s primary limitation is its reliance on a single neural network model developed with Easy NN-Plus software. While the neural network provided useful predictive insights, its complexity poses challenges for interpretability, and its accuracy is dependent on the quality and diversity of the training data. Additionally, this study’s relatively limited sample size and its focus on a single hospital cohort may limit the generalizability of these findings to broader, more diverse patient populations and healthcare settings.

### 5.2. Future Research Directions

Future research should consider testing a range of machine learning models, such as logistic regression, k-nearest neighbors (KNN), naïve Bayes (NB), decision trees (DT), and support vector machines (SVM), to directly compare their predictive accuracy and interpretability with that of the neural network. Such comparisons would help determine the most effective model for predicting complications in trauma patients with lower limb fractures. Expanding the dataset to include data from multiple healthcare centers would further enhance the model’s robustness and generalizability. Additionally, incorporating real-time monitoring data could improve the model’s predictive accuracy, allowing for even earlier detection of high-risk cases and enabling more timely interventions.

## Figures and Tables

**Table 1:** Age distribution of DVTs.

AGE		
N	Valid	51
	Missing	0
Mean		71.65
Median		77
Std. Deviation		16.238
Skewness		−0.785
Std. Error of Skewness		0.333
Kurtosis		−0.258
Std. Error of Kurtosis		0.656
Minimum		32
Maximum		97

**Table 2:** Input data of the neural network.

	Situation	INR	PT	APTT	Activity+	TGO	TGP	Bilirubin+	Bilirubin+
T:0	false	2.03	22.8	33.8	47.9	195	60	3.95	1.82
T:1	false	1.26	15.2	27	72.3	34	44	1.01	0.3
T:2	false	1.04	13.2	23.1	95.1	12	21	0.66	0.3
T:3	false	1.04	12.7	20.7	103	25	19	1.12	0.45
T:4	false	1.43	16.2	27.3	59.64	13	11	1.35	0.7
T:5	false	0.93	11.2	12.6	115.21	28	13	0.89	0.35
T:6	false	1.14	14.2	29.7	62.36	44	24	0.87	0.36
T:7	false	1.24	14.4	21.3	73.13	30	17	0.65	0.33

**Table 3:** Comparative analysis of optimized and normal values.

Status	Parameters	Optimized Values	Values Outside Normal Limits
FALSE	Direct bilirubin	1.633	1.633 > 0.3
	Prothrombin activity	7.25	7.25 < 70
	APTT	19.1	19.1 < 20
	PT	77.72	77.72 > 14
	ALT	5	-
	INR	10.27	10.27 > 1.2
	AST	12	-
	Total bilirubin	0.34	-
TRUE	Direct bilirubin	0.01	-
	Prothrombin activity	112.48	-
	APTT	93.3	93.3 > 40
	PT	10.6	10.6 < 11
	ALT	562.65	562.65 > 40
	INR	0.93	-
	AST	12	-
	Total bilirubin	77.48	77.48 > 1.2

**Table 4:** Optimized data for achieving the “TRUE” status.

Status	Parameter	Optimized Values with Limits
TRUE	Direct bilirubin	0.01
	Prothrombin activity	112.48
	APTT	30
	PT	12
	ALT	20
	INR	0.93
	AST	12
	Total bilirubin	0.5

**Table 5:** Recommendations on thromboprophylaxis according to ACCP VIII.

Major General Surgery Patients	Thromboprophylaxis Until Discharge
Patients at high thrombotic risk, including those undergoing major cancer surgery or with a history of VTE	Thromboprophylaxis after discharge, possibly up to 28 days
Patients undergoing major gynecologic surgery	Thromboprophylaxis up to 28 days
Patients with major orthopedic surgery (hip replacement, knee replacement, surgical femur fracture)	Thromboprophylaxis at least ten days in hospital; extension up to 35 days recommended—grade 1A.

**Table 6:** Wells score for DVT predicting [34].

Wells Score Criteria Description	Points
Active cancer (treatment within the last six months or palliative)	+1
Calf swelling ≥ 3 cm compared to asymptomatic calf (measured 10 cm below tibial tuberosity)	+1
Swollen unilateral superficial veins (non-varicose in the symptomatic leg)	+1
Unilateral pitting edema (in the symptomatic leg)	+1
Previously documented DVT	+1
Swelling of the entire leg	+1
Localized tenderness along the deep venous system	+1
Paralysis, paresis, or recent cast immobilization of lower extremities	+1
Recently bedridden for ≥3 days or major surgery requiring local or general anesthetic in the past 12 weeks	+1
Alternative diagnosis at least as likely	−2

## Data Availability

The dataset is available on request from the authors.

## Abbreviations

DV	deep vein thrombosis
BMI	body mass index
VTE	venous thromboembolism
PE	pulmonary thromboembolism
AIDS	acquired immunodeficiency syndrome
NN	neural network
MB	major bleeding
MRSA	Methicillin-resistant Staphylococcus Aureus
N	number
NOACs	non-vitamin K oral anticoagulants
VKA	vitamin K antagonists
ALT	alanine aminotransferase
AST	aspartate aminotransferase
PT	prothrombin time
INR	international ratio
TGO	glutamate-oxaloacetate-transaminase
TGP	glutamate-pyruvate-transaminase
T	time
